# AKR1B1 as a Prognostic Biomarker of High-Grade Serous Ovarian Cancer

**DOI:** 10.3390/cancers14030809

**Published:** 2022-02-05

**Authors:** Marko Hojnik, Nataša Kenda Šuster, Špela Smrkolj, Damjan Sisinger, Snježana Frković Grazio, Ivan Verdenik, Tea Lanišnik Rižner

**Affiliations:** 1 Institute of Biochemistry, Faculty of Medicine, University of Ljubljana, 1000 Ljubljana, Slovenia; marko.hojnik@mf.uni-lj.si; 2 Department of Pathology, University Medical Centre Maribor, 2000 Maribor, Slovenia; damjan.sisinger@ukc-mb.si; 3 Division of Gynecology, Department of Obstetrics and Gynecology, University Medical Centre Ljubljana, 1000 Ljubljana, Slovenia; natasa.kendasuster@kclj.si (N.K.Š.); spela.smrkolj@mf.uni-lj.si (Š.S.); ivan.verdenik@guest.arnes.si (I.V.); 4 Division of Gynecology and Obstetrics, Medical Faculty, University of Ljubljana, 1000 Ljubljana, Slovenia; 5 Division of Gynecology, Department of Pathology, University Medical Centre Ljubljana, 1000 Ljubljana, Slovenia; snjezana.frkovicgrazio@kclj.si

**Keywords:** high-grade serous ovarian cancer, survival, prognosis, immunohistochemistry, biomarker, aldo-keto reductase family 1 member B1 (AKR1B1), aldo-keto reductase family 1 member B10 (AKR1B10), resistance

## Abstract

**Simple Summary:**

We evaluated the levels of AKR1B1 and AKR1B10 in 99 patients with high-grade serous ovarian cancer and their association with clinicopathological characteristics, survival, and response to chemotherapy. An immunohistochemical analysis showed that higher AKR1B1 levels correlated with a better disease-free survival of patients whereas we saw no differences for AKR1B10 levels. A multivariant Cox analysis identified high AKR1B1 levels as an important prognostic factor for both overall and disease-free survival. A further analysis revealed no association between AKR1B1 and AKR1B10 levels and response to chemotherapy.

**Abstract:**

Although aldo-keto reductases (AKRs) have been widely studied in cancer, no study to date has examined the roles of AKR family 1 members B1 (AKR1B1) and B10 (AKR1B10) in a large group of ovarian cancer patients. AKR1B1 and AKR1B10 play a significant role in inflammation and the metabolism of different chemotherapeutics as well as cell differentiation, proliferation, and apoptosis. Due to these functions, we examined the potential of AKR1B1 and AKR1B10 as tissue biomarkers. We assessed the immunohistochemical levels of AKR1B1 and AKR1B10 in tissue paraffin sections from 99 patients with high-grade serous ovarian cancer (HGSC) and compared these levels with clinicopathological characteristics, survival, and response to chemotherapy. A higher immunohistochemical AKR1B1 expression correlated with a better overall and disease-free survival of HGSC patients whereas AKR1B10 expression did not show any significant differences. A multivariant Cox analysis demonstrated that a high AKR1B1 expression was an important prognostic factor for both overall and disease-free survival. However, AKR1B1 and AKR1B10 were not associated with different responses to chemotherapy. Our data suggest that AKR1B1 is involved in the pathogenesis of HGSC and is a potential prognostic biomarker for this cancer.

## 1. Introduction

High-grade serous ovarian cancer (HGSC) is the most common malignancy of the ovary [1,2,3]. The most likely origin of HGSC is considered to be the epithelium of the fallopian tube fimbriae [3]. The World Health Organization classification of tumors divides serous ovarian cancer into low- and high-grade serous ovarian cancer, which are two etiologically and morphologically distinct entities [1]. The general characteristics of HGSC are a solid, papillary, glandular, or cribriform architecture; sheets of malignant cells with a high mitotic index; enlarged and pleomorphic nuclei; and a *TP53* deleterious mutation frequency of nearly 100% [1,3]. By contrast, low-grade serous ovarian cancer has small nests and glands; complex papillae or micropapillae; low-grade nuclear atypia; and exhibits *KRAS*, *NRAS*, *BRAF*, *USP9X,* and *EIF1AX* mutations [1].

HGSC is subdivided according to the gene expression into four descriptive groups: immunoreactive, differentiated, proliferative, and mesenchymal. However, these groups have not yet been applied diagnostically or clinically [4]. HGSC is characterized by very aggressive tumors and high mortality rates and is usually detected at an advanced stage of the disease (75–80% of cases). Current first-line treatment for HGSC involves cytoreductive surgery followed by chemotherapy, usually carboplatin and paclitaxel [5]. The purpose of primary cytoreductive surgery is to resect all macroscopically visible tumor remnants in the abdominal cavity as well as disease staging. In inoperable cases, patients receive neoadjuvant chemotherapy [6].

After surgery, all HGSC patients undergo adjuvant chemotherapy and most of them achieve remission after the initial treatment. Recurrence of the disease, which is mostly resistant to chemotherapy, usually occurs 18–24 months after the first treatment of the disease [3,7]. So far, the most useful prognostic and predictive biomarkers for HGSC are germline deleterious mutations of *BRCA1* and *BRCA2*. Cancers with these mutations are substantially more susceptible to the class of poly adenosine diphosphate-ribose polymerase (PARP) inhibitors [2]. PARP inhibitors were initially approved only for patients with *BRCA* mutations [6]. However, the Food and Drug Administration later expanded the indications to relapsed ovarian cancer irrespective of the *BRCA* mutation status or platinum sensitivity [8]. Current guidelines from the American Society of Clinical Oncology recommend PARP inhibitors for maintenance therapy for patients with stage III–IV HGSC that is in complete or partial response to first-line platinum-based chemotherapy [9].

Recent studies showed that up to 50% of HGSC have a homologous recombination repair deficiency (HRD) [10,11]. Homologous recombination (HR) is one of the key mechanisms for the repair of double-strand breaks. This complex process involves several genes, including *BRCA1/2* where mutations of these genes lead to HRD. Defects in the repair of DNA breaks result in accumulated mutations, an increased susceptibility to DNA damage, and cell death. HGSC patients with HRD have a significantly prolonged progression-free survival and an increased responsiveness to chemotherapy, especially platinum agents, and PARP inhibitors. Therefore, HRD testing is an important prognostic and predictive biomarker in HGSC [10,11].Another targeted therapy for HGSC uses anti-angiogenic agents (bevacizumab) [8]. Additionally, serine/threonine-specific protein kinase inhibitors (afuresertib) show promising results [8]. 

The mechanisms of chemoresistance have been thoroughly studied in many cancers; however, there have not been any significant breakthroughs in the prevention or treatment of chemoresistant cancers [12,13]. The reported mechanisms of resistance include reduced apoptosis, increased antioxidant production and the detoxification of reactive oxygen species, altered intracellular drug transport, repair of DNA damage, reversion mutations [14,15], and the metabolism of chemotherapeutics to their less effective metabolites [16]. New biomarkers may provide a more accurate and prognostically relevant subclassification of HGSC that might predict survival or response to chemotherapy.

The aldo-keto reductase (AKR) superfamily comprises several enzymes that are involved in important biochemical processes. AKR1B1, AKR1B10, and AKR1B15 are the only three human members of the AKR1B subfamily. These enzymes catalyze the NADPH-dependent reduction of carbonyl groups to hydroxyl groups on different endogenous and exogenous substrates. AKR1B1 catalyzes the reduction of glucose to sorbitol and plays a role in osmoregulation and the polyol pathway. It acts as a prostaglandin PGF2α synthase [17] and indirectly affects the protein kinase C pathway, which stimulates nuclear factor kappa B-induced inflammation and cell proliferation [17,18,19,20]. AKR1B10 catalyzes the reduction of isoprenyl aldehydes, affecting the prenylation of small guanosine triphosphatases (GTPases) and cell proliferation [21]. It also acts as a retinal reductase, which leads to the depletion of retinoic acid that has pro-differentiating effects [22]. AKR1B10 also controls fatty acid biosynthesis, which has important functions in carcinogenesis [23,24]. AKR1B1 and AKR1B10 induce cell resistance to different chemotherapeutics, including cisplatin, daunorubicin, and idarubicin [25,26], and can exert protective actions by detoxifying the products of lipid peroxidation, e.g., cytotoxic carbonyl 4-hydroxynonenal to 4-hydroxynonenol [27].

In this study, we evaluated the potential of AKR1B1 and AKR1B10 as prognostic tissue biomarkers for HGSC by evaluating the immunohistochemical (IHC) levels of AKR1B1 and AKR1B10 in tissue paraffin sections from a large group of well-characterized patients.

## 2. Materials and Methods

### 2.1. Study Groups

The study cohort included 99 patients with HGSC (selected cases were diagnosed from 2002 to 2012). Paraffin-embedded tissue samples of the primary tumors from each patient were obtained from the archive and demographic, clinical, and histopathological data were collected (Table 1). IHC staining of AKR1B1 and AKR1B10 was performed and the results were correlated with the clinicopathological data, including cumulative survival, disease-free survival, stage of disease, and response to chemotherapy.

### 2.2. Immunohistochemistry

IHC was performed for the visualization and localization of specific antigens on formalin-fixed, paraffin-embedded HGSC tissue samples from the archives at the University Medical Centre Ljubljana, Division of Gynecology, Department of Pathology. All the samples were reassessed by a pathologist who morphologically and immunohistochemically confirmed the diagnosis of HGSC before including the case in this study. Each paraffin-embedded tissue block was sectioned with a microtome to obtain 3–5 μm-thick paraffin sections, which were placed onto a glass slide (Superfrost Plus; Thermo Scientific, Leicestershire, UK).

The tissue slides were dehydrated in a slide-drying ventilation oven for 60 min at 60 °C. IHC staining with anti-AKR1B1 and anti-AKR1B10 antibodies was carried out on an automated system (BenchMark Ultra; Ventana, Basel, Switzerland) using detection kits (OptiView DAB; Ventana; Basel, Switzerland; cat. no. 760-700) following the manufacturer’s instructions.

A deparaffinization solution (EZPrep solution; Ventana, Basel, Switzerland; cat. no. 950-102) was used for 4 min at 72 °C for the complete dissolution of the paraffin. A tris-based buffer at pH 8.5 (cell conditioning solution CC1; Ventana, Basel, Switzerland; cat. no. 950-124) was used for the epitope retrieval for AKR1B10 (24 min) and AKR1B1 staining (32 min) at 95 °C. The slides were applied and incubated with the primary antibodies anti-AKR1B1 (Abcam; Cambridge, UK; cat. no. ab62795, lot: GR64780-2) and anti-AKR1B10 (Abcam; Cambridge, UK; cat. no. ab96417, lot: GR13314-31) for 32 min (optimized dilution 1:200 in an antibody diluent (Dako; Agilent, Santa Clara, CA, USA, cat. No. S080983-2)). The positive and negative controls for AKR1B1 and AKR1B10 were normal liver tissue (hepatocytes, ductal epithelium, and connective tissue) (see the validation of antibodies in Table 2). The IHC valuation was based on the proportions (%) of the stained cells. The IHC stained tissue sections were independently assessed and scored by two pathologists (M.H. and D.S.). Inter-observer reproducibility was determined by the interclass correlation coefficient, which was > 0.9.

### 2.3. Statistics

A proportional Cox model analysis was performed to evaluate the survival. First, the percentages of positive AKR1B1 and AKR1B10 cancer cells were used as a continuous variable in a multivariate model. To estimate the non-linear relationship, we used restricted cubic splines (with three knots) with modified AKR1B1 values before entering the Cox proportional hazards (CPH) model. The resultant position of the knots was consequently used as a threshold for using the AKR1B1 value as a dichotomous variable in the Cox model. The restricted cubic splines analysis and the corresponding figures were performed with R version 4.1 [29] using the rms package [30] for restricted cubic splines. All other computation was performed using SPSS Statistics v27 (IBM; Armonk, NY, USA).

The correlations between the percentages of the AKR1B1 and AKR1B10 expression in the cancer cells and other clinical data were evaluated using Mann–Whitney U and Kruskal–Wallis statistical tests using SPSS v27 (IBM, Armonk, NY, USA).

### 2.4. Ethical Issues 

The National Medical Ethics Committee of the Republic of Slovenia (0120-701/2017-6) approved this retrospective study.

## 3. Results

### 3.1. Demographic and Histopathological Characteristics of the Patients 

The study group comprised 99 patients with HGSC, of which 80 patients were diagnosed with FIGO stage III–IV disease (Table 1). The 5-year cumulative survival was 29.3%. The follow-up data from 0.25–12.6 years (median: 3.2 years) were collected for 97 out of 99 patients. All clinical and histopathological data are provided in the Supplementary tables (Tables S1–S3).

### 3.2. AKR1B1 and AKR1B10 Expression Levels in HGSC

**Figure 1:**
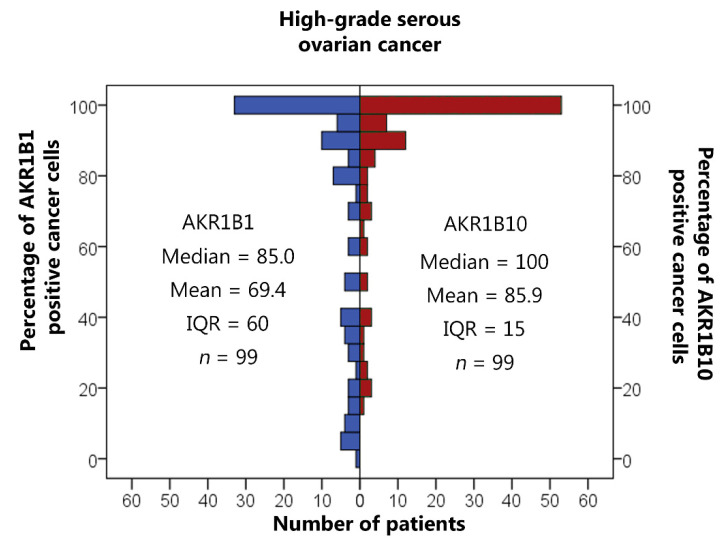
Immunohistochemical staining of AKR1B1 and AKR1B10 in high-grade serous ovarian cancer. The graph shows the numbers of cases with the associated percentages of positive cancer cells. AKR1B1: aldo-keto reductase family 1 member B1; AKR1B10: aldo-keto reductase family 1 member B10; IQR: interquartile range; *n*: number of patients.

**Figure 2:**
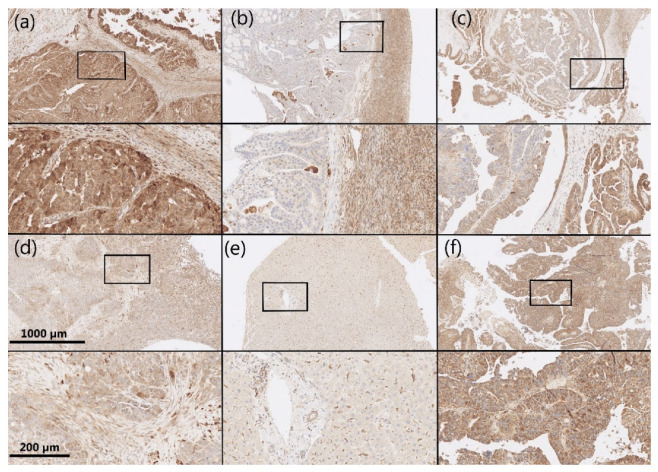
Representative immunohistochemical AKR1B1 staining in HGSC samples and control tissue. (**a**–**d**) HGSC; (**e**) control liver tissue; (**f**) control endometrioid endometrial cancer. Upper half of panels: 50 × magnification; lower half of panels: the framed area from the upper half of the panel (200 × magnification). AKR1B1: aldo-keto reductase family 1 member B1; HGSC: high-grade serous ovarian cancer.

**Figure 3:**
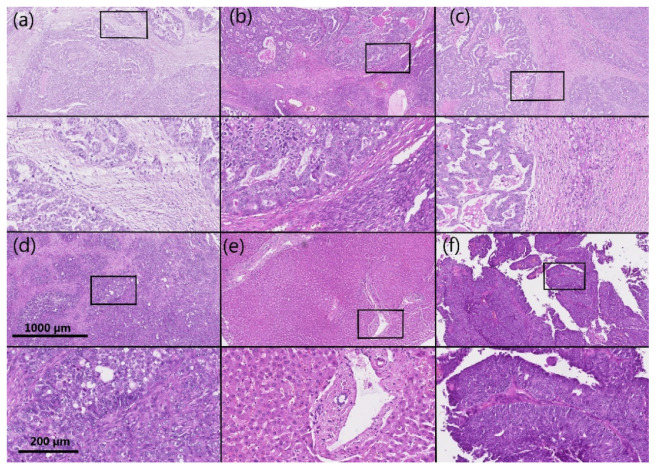
Representative hematoxylin and eosin staining of the same samples as shown in Figure 2. (**a**–**d**) HGSC; (**e**) control liver tissue; (**f**) control endometrioid endometrial cancer. Upper half of panels: 50 × magnification; lower half of panels: the framed area from the upper half of the panel (200 × magnification). HGSC: high-grade serous ovarian cancer.

**Figure 4:**
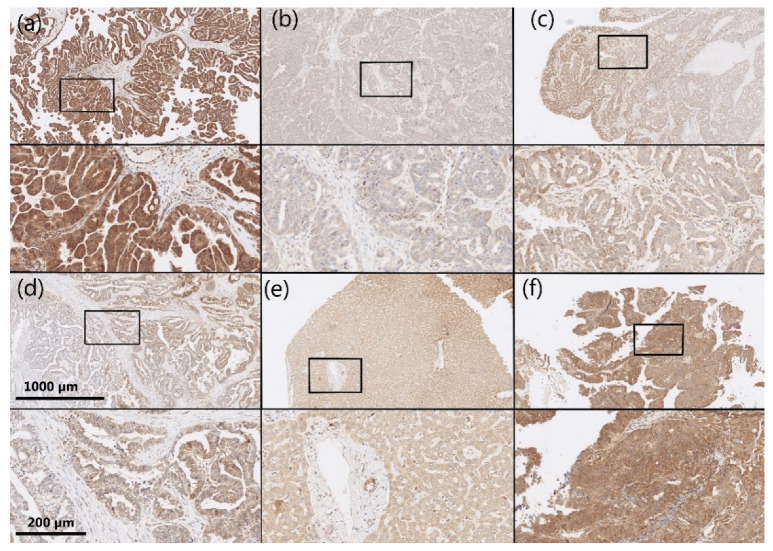
Representative immunohistochemical AKR1B10 staining in HGSC samples and control tissue. (**a**–**d**) HGSC; (**e**) control liver tissue; (**f**) control endometrioid endometrial cancer. Upper half of panels: 50 × magnification; lower half of panels: the framed area from the upper half of the panel (200 × magnification). AKR1B10: aldo-keto reductase family 1 member B10; HGSC: high-grade serous ovarian cancer.

**Figure 5:**
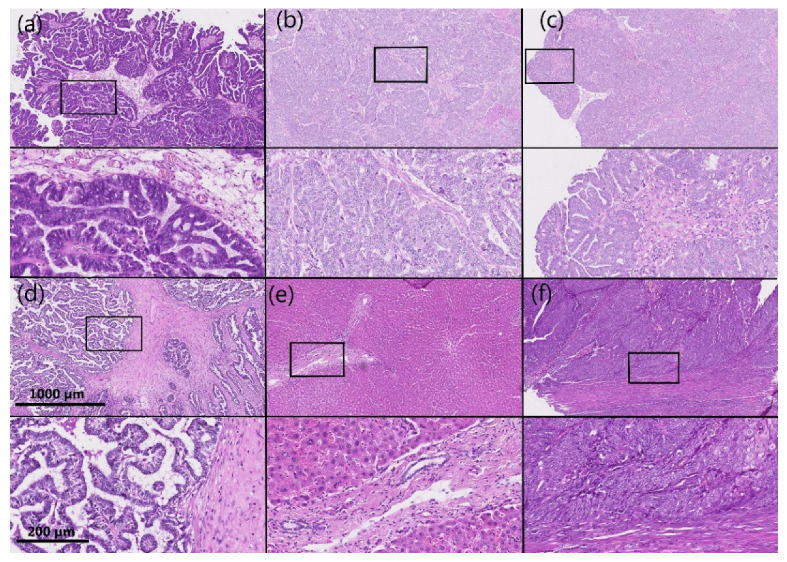
Representative hematoxylin and eosin staining of the same samples as shown in Figure 4. (**a**–**d**) HGSC; (**e**) control liver tissue; (**f**) control endometrioid endometrial cancer. Upper half of panels: 50 × magnification; lower half of panels: the framed area from the upper half of the panel (200 × magnification). HGSC: high-grade serous ovarian cancer.

AKR1B1 and AKR1B10 staining was observed within the cytoplasm and nucleus of the epithelial cancer cells in HGSC as well as in the endothelium and ovarian stroma. The median and mean percentages of AKR1B1-positive cancer cells were 85.0% and 69.4%, respectively (IQR = 60.0, SD = 34.0). The median and mean percentages of AKR1B10-positive cancer cells were 100.0% and 85.9%, respectively (IQR = 15.0, SD = 23.5) (Figure 1). An adjacent ovarian stroma revealed strong positive reactions within the cytoplasm and nucleus. Representative pictures of the immunohistochemical staining and hematoxylin and eosin staining are presented in Figure 2, Figure 3, Figure 4 and Figure 5.

### 3.3. The Correlation between AKR1B1 and AKR1B10 Expression Levels and Survival 

**Figure 6:**
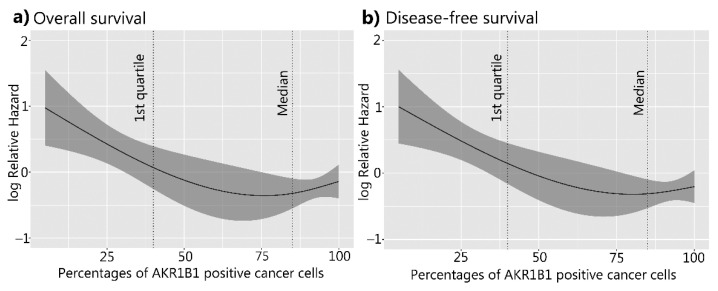
Overall and disease-free survival of patients with HGSC in relation to AKR1B1. (**a**) Overall survival (*p* = 0.006) and (**b**) disease-free survival curves (*p* = 0.002). The X-axis represents the percentages of AKR1B1-positive cancer cells. The Y-axis represents the hazard ratio for death (**a**) and disease relapse (**b**). AKR1B1: aldo-keto reductase family 1 member B1; HGSC: high-grade serous ovarian cancer.

For the survival studies of patients with HGSC, continuous variables of the percentages of AKR1B1- and AKR1B10-positive cancer cells were used as predictors in the Cox survival models. A higher AKR1B1 expression was significantly associated with a better overall survival (*p* = 0.006) and disease-free survival (*p =* 0.002) (Figure 6). A multivariant Cox model analysis identified a higher percentage of AKR1B1-positive cancer cells (using continuous variables) as a statistically important prognostic factor for survival (*p* = 0.01), disease-free survival (*p =* 0.005), and FIGO stage (*p* = 0.01 and *p* = 0.005) (Table 3 and Table 4). Considering that the restricted cubic splines model with three knots (Figure 6) crossed zero around the first quartile, we decided to also calculate the Cox model for the AKR1B1 threshold at the first quartile value (40). The results are provided in the Supplementary data (Figure S3; Tables S6 and S7).

**Figure 7:**
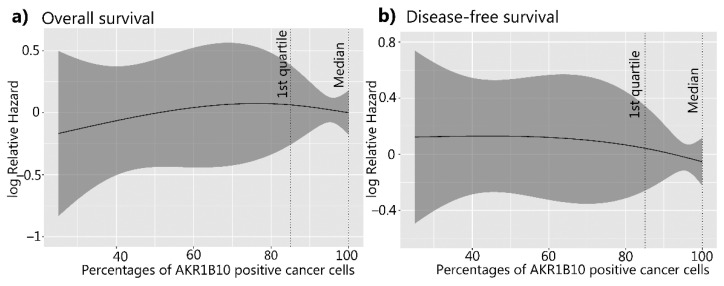
Overall survival and disease-free survival of patients with HGSC in relation to AKR1B10. (**a**) Overall survival (*p* = 0.72) and (**b**) disease-free survival curves (*p* = 0.82). The X-axis represents the percentages of AKR1B10-positive cancer cells. The Y-axis represents the hazard ratio for death (**a**) and disease relapse (**b**). AKR1B10: aldo-keto reductase family 1 member B10; HGSC: high-grade serous ovarian cancer.

The AKR1B10 expression levels did not show any significant differences in the overall or disease-free survival (*p* = 0.72 and *p* = 0.82, respectively) and were not recognized as a prognostic factor for survival (Figure 7; Table 3 and Table 4). 

When the groups were separated into two groups using the median values of the percentages of AKR1B1- or AKR1B10-positive cancer cells as the threshold values, no significant differences were observed. An analysis of both AKR1B1 and AKR1B10 together, using the median value as the separating value, also did not show any statistical difference (Figures S1 and S2; Tables S4 and S5).

Residual disease after primary cytoreductive surgery was also confirmed as a significant factor in the overall and disease-free survival in our patient group (*p* < 0.001) *(*Figure S4). In the group of patients with macroscopic residual disease or no cytoreductive surgery, higher levels of AKR1B1 were associated with a better overall survival (*p =* 0.030) and disease-free survival (*p* = 0.007); the AKR1B10 expression levels did not show any significant association with the overall or disease-free survival (*p =* 0.801 and *p* = 0.479, respectively) (Table S8). In the group of patients with microscopic residual disease, the higher levels of AKR1B1 were not associated with a better overall survival (*p =* 0.099) and disease-free survival (*p =* 0.124); similarly, no association was seen between the AKR1B10 levels and overall or disease-free survival (*p =* 0.083 and *p* = 0.346, respectively) (Table S9).

Our results of the survival studies were also compared with publicly available data from cBioportal (https://www.cbioportal.org accessed on 19 December 2021) and the National Cancer Institute Proteomic Data Commons (PDC) server (https://pdc.cancer.gov) (accessed on 19 December 2021). We used the RNA expression data of the genes *AKR1B1* and *AKR1B10* in the tissues of high-grade serous ovarian cancer (acquired by RNA-Seq and Expectation-Maximization (RSEM) algorithms with batch normalization) and clinical data from the TCGA Pan-Cancer Atlas study [31,32]. The RNA expression levels of *AKR1B1* and *AKR1B10* did not show any significant associations with the overall or disease-free survival (and were not recognized as a prognostic factor for survival (Tables S10 and S11)).

For the analysis of protein levels in HGSC, we used the National Cancer Institute Proteomic Data Commons (PDC) server (https://pdc.cancer.gov Zhang TCGA study) (accessed on 19 December 2021) [33]. Mass-spectrometry-based proteomic data for AKR1B1 and AKR1B10 also failed to reveal a significant association between the protein levels and overall or disease-free survival (Tables S12 and S13).

### 3.4. The Correlation between AKR1B1 and AKR1B10 Expression Levels and Chemoresistance

**Figure 8:**
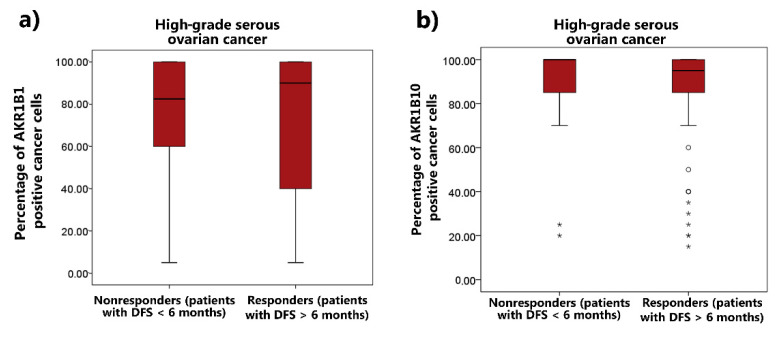
(**a**) AKR1B1 and (**b**) AKR1B10 distributions between patients with HGSC and different responses to chemotherapy. Median values, boxes from the 25th to 75th percentiles, and whiskers that correspond with the 25th percentile minus 1.5 times the interquartile range and with the 75th percentile plus 1.5 times the interquartile range are shown. ° represents mild outliers; * represents extreme outliers; AKR1B1: aldo-keto reductase family 1 member B1; AKR1B10: aldo-keto reductase family 1 member B10; IQR: interquartile range; HGSC: high-grade serous ovarian cancer.

The correlations between the AKR1B1 and AKR1B10 expression and the responses of patients to chemotherapy were also examined using the Mann–Whitney U test. The patients were divided into two groups according to their responses to chemotherapy: (a) non-responders (patients who did not achieve a disease-free survival of 6 months); and (b) responders (patients with a disease-free survival of at least 6 months). No significant differences were observed in the AKR1B1 (*p =* 0.93) and AKR1B10 (*p =* 0.55) expression between the responders and non-responders (Figure 8). We also analyzed the data from the proteomic database/Zhang TCGA study [33]; the results were consistent with our results (Table S14).

## 4. Discussion

Many studies have demonstrated the role of AKRs in the pathogenesis of many different diseases [34], including uterine diseases [35,36,37]. The role of AKR enzymes in cancer has especially become an important topic in the last 40 years, with more than 860 published papers focusing on AKR and cancer indexed in MEDLINE since 1981. AKR1B1 and AKR1B10 have been associated with different cancers [18,37,38,39,40,41,42]; however, their roles differ among cancer types. Their increased expression was associated with either longer or shorter patient survival, depending on the cancer type. AKR1B1 expression is increased in rectal, hepatocellular, lung, breast, cervical, and ovarian cancer [38,43,44,45,46] and decreased in colorectal and endometrial cancer [28,47,48,49]. An increased AKR1B10 expression was associated with a poorer prognosis in oral squamous cells, gastric carcinomas, and lung adenocarcinomas [39,41,50,51] whereas a decreased AKR1B10 expression was associated with a significantly worse survival in colorectal cancer [52].

To the best of our knowledge, only a few studies have evaluated AKR levels in ovarian cancer cells. It was discovered that upregulated mRNA of the AKR1C1–4 enzymes (originally known as dihydrodiol dehydrogenases) and AKR1A1 induces a resistance to cisplatin in human ovarian cancer cells [53]. Other reports have revealed that AKR1B1 protein levels are significantly upregulated in fibroblasts cocultured with ovarian cancer cells [54] and also in ovarian cancer [44]. It was suggested that AKR1B1 overexpression may render cancer cells resistant to anticancer drugs and that AKR1B1 inhibitors could reverse this resistance [55].

Until now, no study has assessed AKR1B1 and AKR1B10 levels using IHC in a large cohort of ovarian cancer patients. Thus, we evaluated the histopathological samples of HGSC patients (*n* = 99) and correlated the AKR1B1 and AKR1B10 expression with the clinicopathological and survival data. Our results revealed that a higher AKR1B1 expression may be associated with a better overall survival of patients with HGSC whereas AKR1B10 expression was not significantly associated with overall or disease-free survival. Additionally, a multivariant Cox analysis demonstrated a high AKR1B1 expression as an important prognostic factor for both overall and disease-free survival. However, these results did not correlate with the survival analysis performed on the RNA expression data and proteomic data from publicly available databases where no association with AKR1B1 was seen. The discrepancies between the analyses of mRNA levels and protein levels could be explained by a variety of factors that affected the translation; methodological approaches (IHC vs. LC-MS/MS after tryptic digest) might lead to different results of survival analyses based on protein AKR1B1 levels.

Surprisingly, AKR1B1 and AKR1B10 were not associated with different responses to chemotherapy although most of the patients included in our study received paclitaxel and carboplatin chemotherapy and both AKR1B1 and AKR1B10 have previously been implicated in the resistance to platinum-based drugs [56]. However, we have to point out that no *BRCA* mutation status or HRD status were available to stratify these patients or to perform a survival analysis accordingly, which represents a weakness of this study.

AKR1B1 and AKR1B10 are involved in many physiological and pathological processes, including inflammation and cell differentiation, proliferation, and apoptosis [34,57]. These actions are achieved by the roles of AKR1B1 and AKR1B10 in retinoid metabolism, prenylation, lipid synthesis, prostaglandin synthesis, and the detoxification of unsaturated carbonyl products of lipid peroxidation [34,35]. Therefore, the potential protective role of AKR1B1 could be explained by its detoxifying function, which decreases oxidative stress and tumor mutations [58,59]. Oxidative stress is recognized by nuclear erythroid 2-related factor 2 (NRF2), which binds to the antioxidant response elements of numerous antioxidant/detoxifying genes, including the AKR genes AKR1B1 and AKR1B10, thus upregulating their expression. Studies that support this explanation showed that NRF2 inducers increase AKR1B1 and AKR1B10 expression and that NRF2 signaling is activated by chemicals that produce reactive oxygen species [60,61,62]. The exact mechanism by which an increased AKR1B1 expression is associated with a better survival of patients with HGSC is currently unknown and requires further studies. It is also unclear why AKR1B10, which plays a protective role in endometrioid endometrial carcinomas [37] and is also induced by NRF2 signaling, does not exert protective effects in HGSC.

Resistance to chemotherapy in HGSC is complex and still not fully understood. There are several mechanisms of resistance, including drug metabolism [16,63], altered drug transport, the suppression of apoptosis [14], reversion mutations [15], the enhancement of DNA repair, and increased antioxidant production and detoxification of reactive oxygen species.Our results showed that AKR1B1 and AKR1B10 levels were not correlated with the response to chemotherapy. This indicated a minor role of AKR1B1 and AKR1B10 in the development of chemoresistance in HGSC.

## 5. Conclusions

In this study, higher levels of AKR1B1 immunostaining were identified as a significant prognostic factor for overall and disease-free survival of patients with HGSC. This indicated an important protective action of AKR1B1. Conversely, AKR1B10 levels showed no correlation with the survival of patients with HGSC. Neither AKR1B1 nor AKR1B10 expression levels correlated with a resistance to chemotherapy. Our data thus suggest that AKR1B1 is involved in the pathogenesis of HGSC but that the exact roles and mechanisms still need to be determined.

## Figures and Tables

**Table 1:** Clinical and histopathological data of HGSC patients.

Characteristic	Detail	Datum
**Age (y)**	Mean ± SD	61.3 ± 11.3
**Ascites (*n* (%))**		54 (54.5)
**Chemotherapy with reported** **follow-up (*n* = 71) (*n* (%))**	Responders (at least 6 months of DFS)	53 (74.6)
	Non-responders (6 months of DFS was not achieved)	18 (25.4)
**Primary chemotherapy (*n*)**	Carboplatin	13
	Docetaxel and carboplatin	4
	Doxorubicin	1
	Gemcitabine	1
	Gemcitabine and carboplatin	1
	Paclitaxel and carboplatin	68
	Paclitaxel and carboplatin and gemcitabine	1
	None	6
	NA	4
**Residual disease after primary chemotherapy (*n*)**	Macroscopic	51
	Microscopic	46
	No cytoreductive surgery	2
**FIGO stage (*n* = 99)**	I–II	19
	III–IV	80
**Grade (*n* = 99)**	High-grade	99
**Follow-up (y)**	Range	0.25–12.6
	Median	3.2
**Five-year survival rate ^a^ (*n* (%))**		29 (29.9)

^a^ Two cases with no follow-up data; *n*: number of patients; SD: standard deviation; y: years; DFS: disease-free survival.

**Table 2:** Antibody description and validation.

Antibody Information					
Antibody	Manufacturer, Catalogue Number, Lot Number	Peptide/ Protein Target	Antigen Sequence	Species Raised, Monoclonal, Polyclonal	Dilution
**Anti-AKR1B1**	Abcam, Cambridge, UK, ab62795, GR64780-2	Aldo-keto reductase family 1 member B1	Aa 300 to the C-terminus (conjugated to keyhole limpet hemocyanin)	Polyclonal rabbit antibody	1:200
**Anti-AKR1B10**	Abcam, Cambridge, UK, ab96417, GR13314-31	Aldo-keto reductase family 1 member B10	Fragment corresponding to aa 1–286	Polyclonal rabbit antibody	1:200
**Antibody Validation**					
Published validation by our research team [28].					
**Current Validation** Positive controls for AKR1B1: Kupffer cells, lymphocytes, endometrioid endometrial cancer cells. Positive controls for AKR1B10: hepatocytes, ductal liver epithelium, lymphocytes, endometrioid endometrial cancer cells. Negative controls for AKR1B1: hepatocytes, fibrous tissue. Negative controls for AKR1B10: fibrous tissue.					

AKR1B1: aldo-keto reductase family 1 member B1; AKR1B10: aldo-keto reductase family 1 member B10.

**Table 3:** Multivariate Cox analysis of independent prognostic factors of overall survival.

Overall Survival	Significance	Hazard Ratio	Confidence Interval
FIGO (I–II vs. III–IV)	*p* = 0.01	2.41	1.23–4.72
Ascites	*p* = 0.80	0.94	0.59–1.50
AKR1B1 expression (continuous variable)	*p* = 0.010	0.991	0.984–0.998
AKR1B10 expression (continuous variable)	*p* = 0.621	1.002	0.993–1.012

**Table 4:** Multivariate Cox analysis of independent prognostic factors of disease-free survival.

Disease-Free Survival	Significance	Hazard Ratio	Confidence Interval
FIGO (I–II vs. III–IV)	*p* = 0.005	2.43	1.31–4.51
Ascites	*p* = 0.761	0.93	0.60–1.46
AKR1B1 expression (continuous variable)	*p* = 0.005	0.990	0.984–0.997
AKR1B10 expression (continuous variable)	*p* = 0.715	0.998	0.989–1.008

## Data Availability

The data presented in this study are available in the Supplementary Materials.

## Supplementary Materials

The following supporting information can be downloaded at: https://www.mdpi.com/article/10.3390/cancers14030809/s1. Figure S1: Overall survival and disease-free survival of patients in relation to AKR1B1; Figure S2: Overall survival and disease-free survival of patients with HGSC in relation to AKR1B10; Figure S3: Overall survival and disease-free survival of patients with HGSC in relation to AKR1B1; Figure S4: Overall survival and disease-free survival of patients with HGSC in relation to residual disease after primary cytoreductive surgery; Table S1: IHC data and clinical data; Table S2: Survival data; Table S3: Chemotherapy data; Table S4: Overall survival analysis for AKR1B1 and AKR1B10 together in patients with HGSC; Table S5: Disease-free survival analysis for AKR1B1 and AKR1B10 together in patients with HGSC; Table S6: Results of multivariant Cox analysis of independent prognostic factors on overall survival (AKR1B1 threshold at value of first quartile (40)); Table S7: Data on multivariant analysis of independent factors predictive of disease-free survival (AKR1B1 threshold at value of first quartile (40)); Table S8: Data on multivariant Cox analysis for overall survival using publicly available RNA and clinical data from the TCGA Pan-Cancer Atlas study; Table S9: Data on multivariant Cox analysis for disease-free survival using publicly available RNA and clinical data from the TCGA Pan-Cancer Atlas study; Table S10: Data on multivariant Cox analysis for overall survival using publicly available proteomic and clinical data from the TCGA study, Zhang et al.; Table S11: Data on multivariant Cox analysis for disease-free survival using publicly available proteomic and clinical data from the TCGA study, Zhang et al.; Table S12: ANOVA analysis using publicly available proteomic and clinical data from the TCGA study, Zhang et al., for AKR1B1 and AKR1B10 protein levels and comparing patients with HGSC and different responses to chemotherapy; Table S13: Overall and disease-free Cox survival analysis for AKR1B1 and AKR1B10 in HGSC patients with macroscopic residual disease after primary cytoreductive surgery; Table S14: Overall and disease-free Cox survival analysis for AKR1B1 and AKR1B10 in HGSC patients with microscopic residual disease after primary cytoreductive surgery.
