# Supplementary material for: AKR1B1 as a Prognostic Biomarker of High-Grade Serous Ovarian Cancer
Source: Cancers (Basel). 2022 Feb 5;14(3):809. doi: 10.3390/cancers14030809 (PMC8834204; doi:10.3390/cancers14030809)
Supplement: Supplementary file 1 [file cancers-14-00809-s001.zip › cancers-1541580-supplementary.pdf]

AKR1B1 as a Prognostic Biomarker of High-grade Serous Ovarian Cancer

Marko Hojnik, Nataša Kenda Šuster, Špela Smrkolj, Damjan Sisinger, Snježana Frković Grazio, Ivan Verdenik and Tea Lanišnik Rižner

Supplement survival analysis.

For survival studies patients with High-grade Serous ovarian cancer (HGSC) were additionally divided into two groups using the median values of percentage of AKR1B1 positive cancer cells (85.0) or AKR1B10 positive cancer cells (100.0) expression as the threshold values. The Kaplan–Meier and Cox survival models were analyzed. As shown by the survival plot (Supplemnet figure S1), no statistically significant differences were found in overall and disease-free survival between the groups above and below the median percentages of AKR1B1 or AKR1B10 positive cancer cells in HGSC (Supplemnet Figure S1 and S2). However, there was a trend for a higher overall survival of patients with percentages of AKR1B1 positive cancer cells above the median score ($p = 0.076$) (Supplemnet Figure S1).

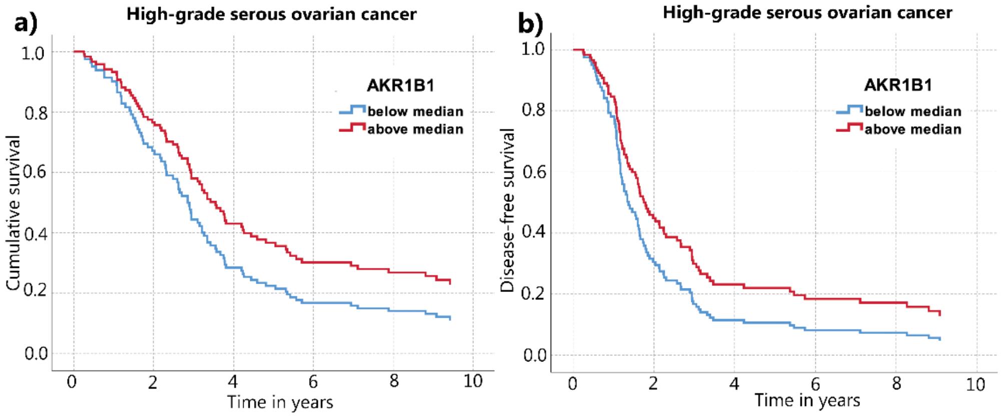

Supplement Figure S1. Overall survival and disease-free survival of patients with HGSC in relation to AKR1B1. One group includes cases with AKR1B1 staining above the median values, and the other group includes cases with AKR1B1 below the median values. (**a**) Overall survival ($p = 0.076$) and (**b**) disease-free survival curves ($p = 0.067$). Time on x axis represents time elapsed since initial diagnosis. AKR1B1: aldo-keto reductase family 1 member B1. HGSC: High-grade ovarian cancer.

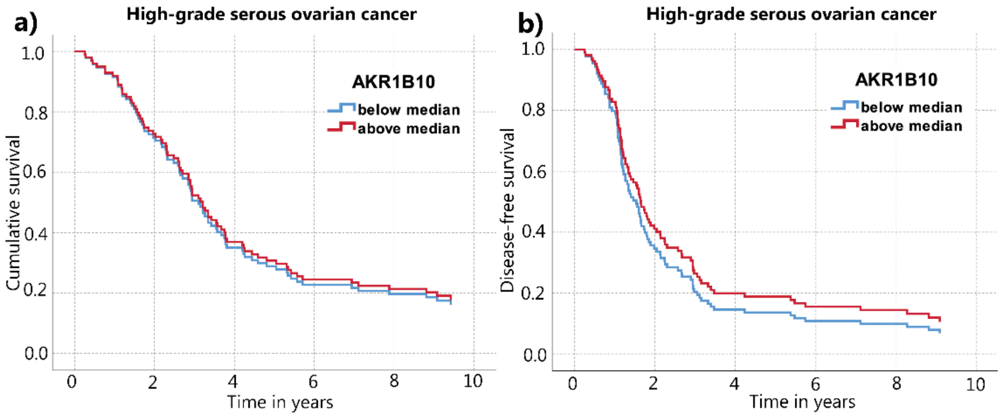

Supplement Figure S2. Overall survival and disease-free survival of patients with HGSC in relation to AKR1B10. One group includes cases with AKR1B10 staining above the median values, and the other group includes cases with AKR1B10 below the median values. (**a**) Overall survival (*p* = *0.82*) and (**b**) disease-free survival curves (*p* = *0.41*). Time on x axis represents time elapsed since initial diagnosis. AKR1B10: aldo-keto reductase family 1 member B10; HGSC: High-grade ovarian cancer.

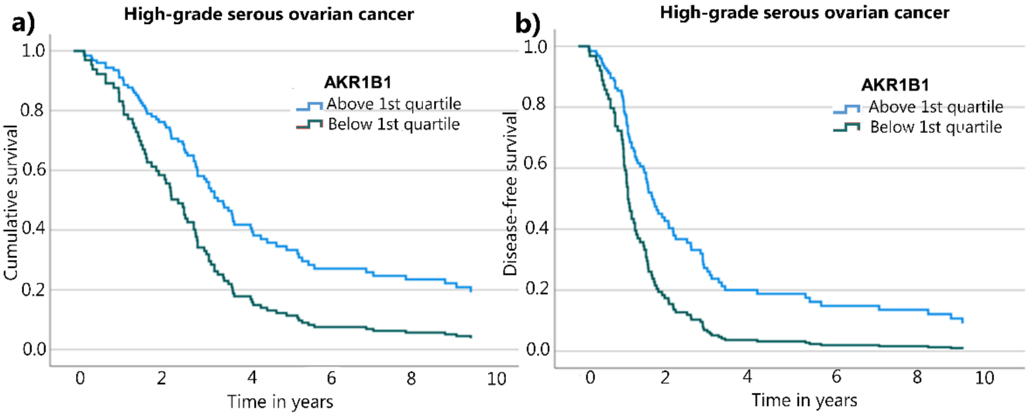

Supplement Figure S3. Overall survival and disease-free survival of patients with HGSC in relation to AKR1B1. One group includes cases with AKR1B1 staining value above the first quartile (40), and the other group includes cases with AKR1B10 below the first quartile (40) value. (**a**) Overall survival (*p* = *0.012*) and (**b**) disease-free survival curves (*p* = *0.005*). Time on x axis represents time elapsed since initial diagnosis. AKR1B1: aldo-keto reductase family 1 member B1; HGSC: High-grade ovarian cancer.

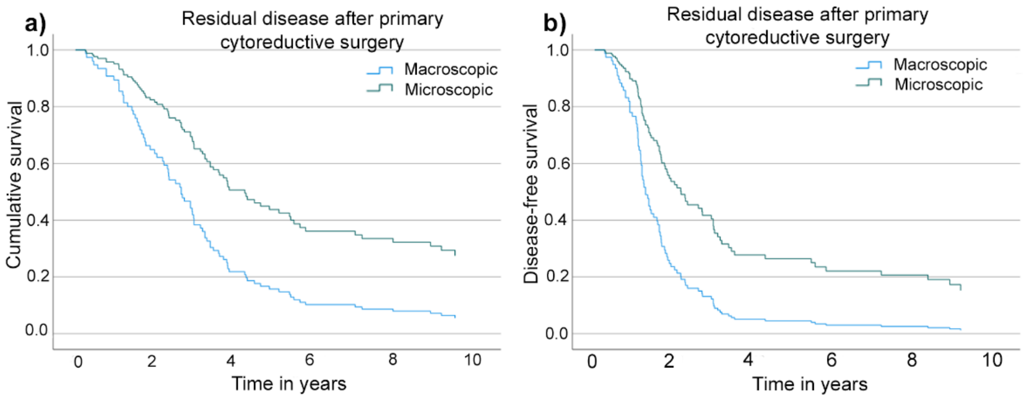

Supplement Figure S4. Overall survival and disease-free survival of patients with HGSC in relation to residual disease after primary cytoreductive surgery. (**a**) Overall survival ($p < 0.001$) and (**b**) disease-free survival curves ($p < 0.001$). Time on x axis represents time elapsed since initial diagnosis. HGSC: High-grade ovarian cancer.

Supplement Table S1. IHC data and clinical data

Case	Carcinoma type	AKR1B1 tumor*	AKR1B10 tumor*	Ascites	Age at diagnosis	FIGO stage
**1**	HGSC	95.0	15.0	Not present	67	III B
**2**	HGSC	85.0	40.0	Present	60	III C
**3**	HGSC	99.0	90.0	Present	69	III A
**4**	HGSC	90.0	20.0	Not present	55	III C
**5**	HGSC	99.0	100.0	Present	80	III C
**6**	HGSC	100.0	85.0	Present	58	III C
**7**	HGSC	100.0	95.0	Present	65	III C
**8**	HGSC	100.0	100.0	Not present	61	I A
**9**	HGSC	100.0	70.0	Present	52	III C
**10**	HGSC	5.0	95.0	Present	40	III B
**11**	HGSC	30.0	100.0	Present	41	III A
**12**	HGSC	100.0	100.0	Not present	56	III A
**13**	HGSC	35.0	35.0	Not present	49	III C
**14**	HGSC	80.0	100.0	Present	61	IV
**15**	HGSC	15.0	100.0	Not present	83	III C
**16**	HGSC	80.0	100.0	Present	72	II C
**17**	HGSC	10.0	95.0	Present	39	III C
**18**	HGSC	100.0	100.0	Not present	72	III C
**19**	HGSC	40.0	100.0	Present	77	III C
**20**	HGSC	100.0	100.0	Not present	58	III C
**21**	HGSC	35.0	100.0	Not present	46	III C
**22**	HGSC	35.0	100.0	Present	51	III C
**23**	HGSC	100.0	100.0	Present	72	IIIC
**24**	HGSC	95.0	100.0	Not present	86	II C
**25**	HGSC	80.0	100.0	Present	69	III C
**26**	HGSC	100.0	100.0	Not present	78	III C
**27**	HGSC	100.0	70.0	Present	50	II B
**28**	HGSC	25.0	100.0	Present	54	III C
**29**	HGSC	60.0	90.0	Not present	40	I C
**30**	HGSC	80.0	50.0	Present	45	III A

**31**	HGSC	90.0	100.0	Not present	55	I C
**32**	HGSC	100.0	90.0	Not present	66	III C
**33**	HGSC	60.0	75.0	Present	56	III C
**34**	HGSC	5.0	90.0	Present	61	III C
**35**	HGSC	80.0	100.0	Not present	61	II C
**36**	HGSC	100.0	85.0	Not present	69	III A
**37**	HGSC	60.0	100.0	Present	77	III C
**38**	HGSC	90.0	100.0	Not present	47	I A
**39**	HGSC	40.0	100.0	Present	62	III C
**40**	HGSC	10.0	100.0	Not present	63	III C
**41**	HGSC	70.0	100.0	Present	63	III C
**42**	HGSC	100.0	100.0	Not present	87	I B
**43**	HGSC	95.0	100.0	Present	64	III B
**44**	HGSC	100.0	100.0	Not present	54	II C
**45**	HGSC	100.0	100.0	Present	67	III C
**46**	HGSC	90.0	25.0	Present	68	III C
**47**	HGSC	90.0	75.0	Present	59	III C
**48**	HGSC	90.0	85.0	Present	42	III C
**49**	HGSC	40.0	100.0	Present	66	III C
**50**	HGSC	20.0	70.0	Not present	70	I C
**51**	HGSC	50.0	20.0	Not present	60	II C
**52**	HGSC	90.0	100.0	Present	66	III C
**53**	HGSC	95.0	90.0	Present	67	III C
**54**	HGSC	100.0	100.0	Present	56	III B
**55**	HGSC	100.0	100.0	Present	65	III C
**56**	HGSC	15.0	60.0	Present	84	III C
**57**	HGSC	50.0	90.0	Not present	82	III C
**58**	HGSC	30.0	40.0	Not present	59	III B
**59**	HGSC	70.0	90.0	Present	61	III C
**60**	HGSC	100.0	100.0	Not present	61	I A
**61**	HGSC	70.0	95.0	Not present	63	IV
**62**	HGSC	90.0	90.0	Present	58	IV
**63**	HGSC	100.0	100.0	Present	58	III C

**64**	HGSC	5.0	65.0	Present	42	III C
**65**	HGSC	100.0	95.0	Present	54	III C
**66**	HGSC	100.0	100.0	Not present	70	I C
**67**	HGSC	95.0	100.0	Present	52	III C
**68**	HGSC	40.0	100.0	Not present	53	III C
**69**	HGSC	100.0	100.0	Not present	55	III B
**70**	HGSC	35.0	20.0	Not present	76	III C
**71**	HGSC	100.0	90.0	Present	55	III C
**72**	HGSC	100.0	100.0	Present	78	III B
**73**	HGSC	85.0	25.0	Not present	56	II C
**74**	HGSC	85.0	100.0	Present	33	III C
**75**	HGSC	5.0	95.0	Not present	46	III C
**76**	HGSC	50.0	90.0	Present	68	III C
**77**	HGSC	100.0	60.0	Not present	65	III C
**78**	HGSC	100.0	100.0	Not present	62	III C
**79**	HGSC	80.0	100.0	Not present	61	III C
**80**	HGSC	40.0	100.0	Present	66	II C
**81**	HGSC	100.0	100.0	Not present	64	I C
**82**	HGSC	90.0	95.0	Not present	70	III C
**83**	HGSC	15.0	100.0	Not present	74	III C
**84**	HGSC	20.0	30.0	Not present	60	I A
**85**	HGSC	80.0	100.0	Present	44	III C
**86**	HGSC	75.0	100.0	Present	71	III C
**87**	HGSC	90.0	90.0	Not present	67	III C
**88**	HGSC	100.0	100.0	Not present	58	III B
**89**	HGSC	10.0	40.0	Present	62	III C
**90**	HGSC	0.0	85.0	Not present	43	III B
**91**	HGSC	20.0	90.0	Present	62	IV
**92**	HGSC	100.0	100.0	Present	50	III C
**93**	HGSC	10.0	80.0	Present	70	III C
**94**	HGSC	100.0	100.0	Not present	65	I C
**95**	HGSC	30.0	100.0	Not present	48	III C
**96**	HGSC	50.0	50.0	Present	61	III C

**97**	HGSC	100.0	100.0	Present	57	III B
**98**	HGSC	5.0	80.0	Not present	80	III C
**99**	HGSC	95.0	100.0	Present	65	IV

*Percentage of positive cancer cell

Supplement Table S2. Survival data

Case	Event death	Event disease relapse	Survival (in years since initial diagnosis)	Time to relapse (in years since initial diagnosis)
**1**	Yes	Yes	3.81	1.46
**2**	Yes	Yes	2.59	1.17
**3**	No	Yes	12.60	2.13
**4**	Yes	Yes	0.95	0.59
**5**	Yes	Yes	1.29	1.34
**6**	Yes	NA	1.07	NA
**7**	Yes	Yes	2.48	1.60
**8**	Yes	Yes	6.94	3.11
**9**	Yes	Yes	2.70	0.67
**10**	Yes	Yes	3.75	1.31
**11**	Yes	Yes	3.21	1.37
**12**	Yes	Yes	3.42	2.95
**13**	Yes	Yes	3.26	0.87
**14**	Yes	Yes	3.78	1.98
**15**	Yes	NA	1.56	NA
**16**	Yes	NA	7.11	NA
**17**	Yes	NA	1.60	NA
**18**	Yes	Yes	5.05	2.04
**19**	Yes	NA	1.17	NA
**20**	Yes	Yes	7.88	5.47
**21**	No	Yes	12.10	5.37
**22**	Yes	Yes	1.43	1.26
**23**	Yes	NA	1.85	NA
**24**	Yes	NA	4.23	NA
**25**	Yes	NA	9.08	NA
**26**	Yes	Yes	2.28	2.14
**27**	No	Yes	11.50	5.74

**28**	Yes	Yes	1.74	0.84
**29**	No	No	11.40	11.40
**30**	Yes	Yes	9.42	1.64
**31**	No	No	11.40	11.40
**32**	Yes	Yes	3.10	1.02
**33**	Yes	Yes	1.19	0.87
**34**	Yes	Yes	1.19	1.14
**35**	Yes	NA	1.08	NA
**36**	Yes	Yes	5.71	3.32
**37**	Yes	NA	0.76	NA
**38**	Yes	Yes	4.44	0.62
**39**	NA	NA	NA	NA
**40**	Yes	Yes	3.67	1.06
**41**	Yes	Yes	1.53	0.71
**42**	No	Yes	11.10	0.56
**43**	Yes	Yes	2.16	1.15
**44**	No	No	11.00	11.00
**45**	Yes	Yes	5.31	3.39
**46**	Yes	Yes	3.55	1.75
**47**	No	No	10.90	10.90
**48**	No	Yes	10.80	8.27
**49**	No	Yes	10.80	1.26
**50**	Yes	Yes	3.34	2.25
**51**	No	Yes	10.80	2.67
**52**	Yes	NA	0.25	NA
**53**	Yes	Yes	4.26	2.66
**54**	Yes	NA	2.94	NA
**55**	Yes	NA	8.81	NA
**56**	Yes	NA	0.27	NA
**57**	Yes	NA	0.55	NA
**58**	Yes	Yes	4.20	1.65
**59**	Yes	Yes	2.62	1.79
**60**	Yes	NA	2.94	NA

**61**	Yes	Yes	5.57	3.47
**62**	Yes	Yes	2.65	1.13
**63**	Yes	Yes	1.69	1.40
**64**	Yes	NA	0.42	NA
**65**	Yes	Yes	2.85	1.66
**66**	Yes	Yes	1.48	1.89
**67**	No	No	9.50	9.50
**68**	Yes	Yes	2.31	1.54
**69**	No	No	9.40	9.40
**70**	Yes	Yes	1.72	1.21
**71**	Yes	Yes	1.64	1.17
**72**	No	Yes	9.30	0.51
**73**	No	NA	9.30	NA
**74**	Yes	NA	2.89	NA
**75**	Yes	Yes	2.90	0.87
**76**	Yes	Yes	2.62	2.57
**77**	Yes	Yes	1.96	1.10
**78**	Yes	Yes	2.32	1.05
**79**	Yes	Yes	3.57	0.93
**80**	Yes	Yes	5.42	3.05
**81**	No	No	9.00	9.00
**82**	Yes	Yes	4.81	2.24
**83**	Yes	NA	1.08	NA
**84**	Yes	Yes	3.34	1.19
**85**	Yes	Yes	2.19	1.22
**86**	Yes	NA	0.76	NA
**87**	Yes	Yes	5.34	1.73
**88**	Yes	Yes	2.93	1.62
**89**	Yes	Yes	2.85	1.34
**90**	Yes	NA	0.45	NA
**91**	NA	NA	NA	NA
**92**	Yes	Yes	2.02	1.14
**93**	Yes	NA	3.15	NA

**94**	Yes	NA	2.30	NA
**95**	Yes	Yes	4.59	1.81
**96**	No	No	8.10	8.10
**97**	Yes	Yes	3.77	2.97
**98**	Yes	Yes	1.40	1.03
**99**	Yes	Yes	3.21	1.07

NA: not available.

Supplement Table S3. Chemotherapy data

Case	Chemotherapy response	Primary chemotherapy	Total lines of chemotherapy	Residual disease after primary cytoreductive surgery
1	Responder	Paclitaxel and Carboplatin	2	Macroscopic
2	Responder	Paclitaxel and Carboplatin	1	Macroscopic
3	Responder	Paclitaxel and Carboplatin	2	Microscopic
4	Non-responder	Paclitaxel and Carboplatin	1	Macroscopic
5	Responder	Carboplatin	2	Macroscopic
6	Non-responder	Paclitaxel and Carboplatin	1	Macroscopic
7	Responder	Paclitaxel and Carboplatin	2	Macroscopic
8	Responder	Carboplatin	1	Microscopic
9	Non-responder	Paclitaxel and Carboplatin	1	Macroscopic
10	Responder	Paclitaxel and Carboplatin	1	Macroscopic
11	Responder	Paclitaxel and Carboplatin	2	Microscopic
12	Responder	Paclitaxel and Carboplatin	2	Microscopic
13	Responder	Paclitaxel and Carboplatin	1	Macroscopic
14	Responder	Doxorubicin	2	Macroscopic
15	NA	NA	NA	Microscopic
16	NA	Carboplatin	1	Microscopic
17	Non-responder	Paclitaxel and Carboplatin	1	Macroscopic
18	Responder	Carboplatin	2	Macroscopic
19	NA	Carboplatin	1	Macroscopic
20	Responder	Paclitaxel and Carboplatin	1	Microscopic
21	Responder	Paclitaxel and Carboplatin	1	Macroscopic
22	Responder	Paclitaxel and Carboplatin	2	Macroscopic
23	NA	None	Non	Macroscopic
24	NA	None	Non	Microscopic
25	NA	Paclitaxel and Carboplatin	1	Macroscopic
26	Responder	Carboplatin	1	Macroscopic
27	Responder	Paclitaxel and Carboplatin	2	Microscopic
28	Non-responder	Paclitaxel and Carboplatin	2	Macroscopic
29	NA	Paclitaxel and Carboplatin	1	Microscopic

30	Responder	Paclitaxel and Carboplatin	3	Microscopic
31	NA	Paclitaxel and Carboplatin	1	Microscopic
32	Responder	Paclitaxel and Carboplatin	2	Macroscopic
33	Non-responder	Paclitaxel and Carboplatin	1	Macroscopic
34	Responder	Paclitaxel and Carboplatin	1	Macroscopic
35	Non-responder	Paclitaxel and Carboplatin	2	Microscopic
36	Responder	Paclitaxel and Carboplatin and Gemcitabine	1	Microscopic
37	Non-responder	Carboplatin	1	Macroscopic
38	NA	None	Non	Microscopic
39	NA	NA	NA	Macroscopic
40	Responder	Paclitaxel and Carboplatin	3	Macroscopic
41	Non-responder	Paclitaxel and Carboplatin	1	Macroscopic
42	Non-responder	Carboplatin	1	Microscopic
43	Responder	Carboplatin	2	Macroscopic
44	NA	Paclitaxel and Carboplatin	1	Microscopic
45	Responder	Gemcitabine and carboplatin	2	Macroscopic
46	Responder	Paclitaxel and Carboplatin	3	Macroscopic
47	NA	Paclitaxel and Carboplatin	1	Macroscopic
48	Responder	Paclitaxel and Carboplatin	2	Macroscopic
49	Responder	Paclitaxel and Carboplatin	3	Microscopic
50	Responder	Paclitaxel and Carboplatin	1	Microscopic
51	Responder	Paclitaxel and Carboplatin	1	Microscopic
52	NA	None	Non	Macroscopic
53	Responder	Paclitaxel and Carboplatin	2	Macroscopic
54	Non-responder	Paclitaxel and Carboplatin	1	Macroscopic
55	Non-responder	Carboplatin	2	Macroscopic
56	NA	Carboplatin	NA	Macroscopic
57	NA	NA	NA	Macroscopic
58	Responder	Paclitaxel and Carboplatin	1	Macroscopic
59	Responder	Paclitaxel and Carboplatin	1	Microscopic
60	NA	Paclitaxel and Carboplatin	1	Microscopic
61	Responder	Paclitaxel and Carboplatin	1	Microscopic
62	Responder	Paclitaxel and Carboplatin	1	Microscopic

63	Responder	Paclitaxel and Carboplatin	1	Microscopic
64	NA	NA	NA	No cytoreductive surgery
65	Responder	Paclitaxel and Carboplatin	1	Microscopic
66	Responder	Carboplatin	1	Macroscopic
67	NA	Paclitaxel and Carboplatin	1	Microscopic
68	Responder	Paclitaxel and Carboplatin	1	Microscopic
69	NA	Paclitaxel and Carboplatin	1	Microscopic
70	Responder	Carboplatin	1	Macroscopic
71	Non-responder	Gemcitabine	1	Macroscopic
72	Non-responder	Paclitaxel and Carboplatin	1	Macroscopic
73	Non-responder	Paclitaxel and Carboplatin	1	Microscopic
74	NA	Paclitaxel and Carboplatin	1	Microscopic
75	Non-responder	Paclitaxel and Carboplatin	1	Macroscopic
76	Responder	Paclitaxel and Carboplatin	1	Microscopic
77	Responder	Paclitaxel and Carboplatin	1	Microscopic
78	Responder	Paclitaxel and Carboplatin	1	Microscopic
79	Non-responder	Paclitaxel and Carboplatin	1	Microscopic
80	Responder	Paclitaxel and Carboplatin	2	Microscopic
81	NA	Paclitaxel and Carboplatin	1	Microscopic
82	Responder	Paclitaxel and Carboplatin	1	Macroscopic
83	Non-responder	Docetaxel and carboplatin	1	Macroscopic
84	Responder	Docetaxel and carboplatin	1	Microscopic
85	Responder	Docetaxel and carboplatin	2	Microscopic
86	NA	Paclitaxel and Carboplatin	1	Macroscopic
87	Responder	Paclitaxel and Carboplatin	1	Macroscopic
88	Responder	Paclitaxel and Carboplatin	1	Microscopic
89	Responder	Paclitaxel and Carboplatin	1	Macroscopic
90	NA	None	None	Macroscopic
91	NA	Paclitaxel and Carboplatin	1	Microscopic
92	Responder	Paclitaxel and Carboplatin	2	Macroscopic
93	NA	Paclitaxel and Carboplatin	1	Microscopic
94	NA	Docetaxel and carboplatin	1	Microscopic
95	Responder	Paclitaxel and Carboplatin	2	Microscopic

96	NA	Paclitaxel and Carboplatin	1	Microscopic
97	Responder	Paclitaxel and Carboplatin	2	Microscopic
98	NA	None	None	Macroscopic
99	Responder	Paclitaxel and Carboplatin	1	No cytoreductive surgery

Responders (patients with disease-free survival of at least six months)
Non-responders (patients who did not achieve six month disease-free survival), NA: not available.

Supplement Table S4. Overall survival analysis for AKR1B1 and AKR1B10 together in patients with HGSC.

			95,0% confidence interval	
	Significance	Hazard ratio	Lower	Upper
**Both AKR1B1 and AKR1B10 > median (reference)**	0.35	1		
**AKR1B1 > median and AKR1B10 < median**	0.83	1.08	0.56	2.06
**AKR1B1 < median and AKR1B10 > median**	0.12	1.61	0.88	2.93
**Both AKR1B1 and AKR1B10 < median**	0.20	1.47	0.82	2.64

AKR1B1: aldo-keto reductase family 1 member B1; AKR1B10: aldo-keto reductase family 1 member B10; HGSC: High-grade ovarian cancer.

Supplement Table S5. Disease-free survival analysis for AKR1B1 and AKR1B10 together in patients with HGSC.

			95,0% confidence interval	
	Significance	Hazard ratio	Lower	Upper
**Both AKR1B1 and AKR1B10 > median (reference)**	0.16	1		
**AKR1B1 > median and AKR1B10 < median**	0.20	1.49	0.81	2.74
**AKR1B1 < median and AKR1B10 > median**	0.03	1.90	1.07	3.39
**Both AKR1B1 and AKR1B10 < median**	0.11	1.58	0.90	2.80

AKR1B1: aldo-keto reductase family 1 member B1; AKR1B10: aldo-keto reductase family 1 member B10; HGSC: High-grade ovarian cancer.

Supplement Table S6. Results of multivariant Cox analysis of independent prognostic factors on overall survival (AKR1B1 threshold at value of first quartile (40)).

**Overall Survival**	Significance	Hazard Ratio	Confidence Interval
**FIGO (I–II vs. III–IV)**	*p* = *0.020*	2.25	1.14–4.44
**Ascites**	*p* = *0.513*	1.17	0.73–1.90
**AKR1B1 expression (< the first quartile value)**	*p* = *0.012*	0.506	0.297–0.860

AKR1B1: aldo-keto reductase family 1 member B1

Supplement Table S7. Data on multivariant Cox analysis of independent factors predictive of disease-free survival (AKR1B1 threshold at value of first quartile (40)).

**Disease-free survival**	Significance	Hazard Ratio	Confidence Interval
**FIGO (I–II vs. III–IV)**	*p* = *0.013*	2.20	1.18–4.10
**Ascites**	*p* = *0.891*	1.03	0.66–1.63
**AKR1B1 expression (< the first quartile value)**	*p* = *0.005*	0.486	0.293–0.808

AKR1B1: aldo-keto reductase family 1 member B1

Supplement Table S8. Data on multivariant Cox analysis for overall survival using publicly available RNA and clinical data from TCGA, PanCancer Atlas study.

**Overall survival**	Significance	Hazard Ratio
**AKR1B1 mRNA levels**	*p = 0.817*	1.000
**AKR1B10 mRNA levels**	*p = 0.561*	1.001
**Diagnosis Age**	*p = 0.017*	1.023

AKR1B1: aldo-keto reductase family 1 member B1; AKR1B10: aldo-keto reductase family 1 member B10.

Supplement Table S9. Data on multivariant Cox analysis for disease-free survival using publicly available RNA and clinical data from TCGA, PanCancer Atlas study

**Disease-free Survival**	**Significance**	**Hazard Ratio**
**AKR1B1 mRNA levels**	*p = 0.390*	1.000
**AKR1B10 mRNA levels**	*p = 0.708*	1.001
**Diagnosis Age**	*p = 0.461*	1.006

AKR1B1: aldo-keto reductase family 1 member B1; AKR1B10: aldo-keto reductase family 1 member B10.

Supplement Table S10. Data on multivariant Cox analysis for overall survival using publicly available proteomic and clinical data from TCGA study Zhang et al.

**Overall Survival**	**Significance**	**Hazard Ratio**
**AKR1B1 protein levels**	*p = 0.956*	1.017
**AKR1B10 protein levels**	*p = 0.891*	0.985
**Diagnosis Age**	*p = 0.068*	1.023

AKR1B1: aldo-keto reductase family 1 member B1; AKR1B10: aldo-keto reductase family 1 member B10.

Supplement Table S11. Data on multivariant Cox analysis for disease-free survival using publicly available proteomic and clinical data from TCGA study Zhang et al.

Disease-free Survival	Significance	Hazard Ratio
**AKR1B1 protein levels**	*p = 0.684*	0.902
**AKR1B10 protein levels**	*p = 0.312*	0.891
**Diagnosis Age**	*p = 0.220*	1.014

AKR1B1: aldo-keto reductase family 1 member B1; AKR1B10: aldo-keto reductase family 1 member B10.

Supplement Table S12. ANOVA analysis using publicly available proteomic and clinical data from TCGA study Zhang et al. for AKR1B1 and AKR1B10 protein levels and comparing patients with HGSC and different responses to chemotherapy

Difference between protein levels in group with responders and non-responders to chemotherapy	Significance
**AKR1B1**	*p = 0.699*
**AKR1B10**	*p = 0.590*

AKR1B1: aldo-keto reductase family 1 member B1; AKR1B10: aldo-keto reductase family 1 member B10; HGSC: High-grade ovarian cancer.

Supplement Table S13. Overall and Disease-free COX survival analysis for AKR1B1 and AKR1B10 in HGSC patients with macroscopic residual disease after primary cytoreductive surgery

			95,0% confidence interval	
**Overall survival**	**Significance**	**Hazard ratio**	**Lower**	**Upper**
**AKR1B1**	0.030	0.991	0.983	0.999
**AKR1B10**	0.801	0.999	0.987	1.010
**Disease-free survival**	**Significance**	**Hazard ratio**	**Lower**	**Upper**
**AKR1B1**	0.007	0.989	0.980	0.997
**AKR1B10**	0.479	0.996	0.985	1.007

AKR1B1: aldo-keto reductase family 1 member B1; AKR1B10: aldo-keto reductase family 1 member B10; HGSC: High-grade ovarian cancer.

Supplement Table S14. Overall and Disease-free COX survival analysis for AKR1B1 and AKR1B10 in HGSC patients with microscopic residual disease after primary cytoreductive surgery

			95,0% confidence interval	
**Overall survival**	**Significance**	**Hazard ratio**	**Lower**	**Upper**
**AKR1B1**	0.099	0.989	0-977	1.002
**AKR1B10**	0.083	1.018	0.998	1.039
**Disease-free survival**	**Significance**	**Hazard ratio**	**Lower**	**Upper**
**AKR1B1**	0.124	0.991	0.979	1.003
**AKR1B10**	0.346	1.008	0.991	1.026

AKR1B1: aldo-keto reductase family 1 member B1; AKR1B10: aldo-keto reductase family 1 member B10; HGSC: High-grade ovarian cancer.
